# Resumption of Direct Oral Anticoagulants After Gastrointestinal Bleeding in Atrial Fibrillation: A Systematic Review of Timing and Clinical Outcomes

**DOI:** 10.7759/cureus.114253

**Published:** 2026-08-10

**Authors:** Bader Jad Allah, Abbas Haider, Sabah Shakeel Shaikh, Kalyan Botsa, Aisha Quadir, Roshna Devi

**Affiliations:** 1 Internal Medicine, California Institute of Behavioral Neurosciences and Psychology, Fairfield, USA; 2 Family Medicine, Abu Dhabi Health Services, Abu Dhabi, ARE; 3 Internal Medicine, Ras Al Khaimah Medical and Health Sciences University, Ras Al Khaima, ARE; 4 Opthalmology, California Institute of Behavioral Neurosciences and Psychology, Fairfield, USA; 5 Diabetes and Endocrinology, California Institute of Behavioral Neurosciences and Psychology, Fairfield, USA; 6 Internal Medicine, Sri Ramachandra Institute of Higher Education and Research, Chennai, IND; 7 Internal Medicine/Family Medicine, California Institute of Behavioral Neurosciences and Psychology, Fairfield, USA

**Keywords:** anticoagulation resumption, atrial fibrillation, direct oral anticoagulants, gastrointestinal bleeding, systematic review, thromboembolism

## Abstract

Gastrointestinal bleeding (GIB) during direct oral anticoagulant (DOAC) therapy poses a clinical dilemma in atrial fibrillation (AF): stopping anticoagulation withdraws stroke protection, while restarting risks hemorrhage. No randomized trials exist, and current AF guidelines offer no fixed restart interval. This review synthesized observational evidence on DOAC resumption timing after GIB in adults with AF.

Six databases were searched (MEDLINE, PubMed, Scopus, Google Scholar, ScienceDirect, and MDPI; January 2015 to April 2026) following the Preferred Reporting Items for Systematic Reviews and Meta-Analyses (PRISMA) 2020 guidelines. Rayyan AI supported deduplication and screening. Risk of bias was assessed using Assessment of Multiple Systematic Reviews 2 (AMSTAR-2), Risk of Bias in Non-randomized Studies of Interventions version 2 (ROBINS-I V2), and the Joanna Briggs Institute (JBI) case report checklist. The outcomes were recurrent GIB, thromboembolic events, and all-cause mortality. Heterogeneity precluded meta-analysis; findings were synthesized narratively.

Ten studies (2018-2025; 68,611 patients with AF across all included cohorts) included two systematic reviews and meta-analyses and eight primary studies. Resumption intervals ranged from under seven days to several months. At the drug-class level, DOAC resumption was not significantly associated with higher recurrent GIB risk in either pooled analysis (odds ratio (OR) 1.09, 95% confidence interval (CI) 0.77-1.53; hazard ratio (HR) 1.22, 95% CI 0.88-1.71); rivaroxaban was the only agent with a significantly elevated rebleeding hazard (HR 1.67, 95% CI 1.16-2.65). Thromboembolic events were 44% to 66% lower among resumers in the two largest cohorts. All-cause mortality was 46 to 50% lower in adjusted models, with comparable reductions in a network meta-analysis of 59,244 patients. The strongest predictor of recurrent hemorrhage was prior GIB; renal impairment and concurrent antiplatelet therapy also raised the risk.

DOAC resumption after GIB in AF was consistently associated with lower thromboembolism and mortality, with no class-level increase in recurrent bleeding. Direct comparisons of resumption timing were too few to identify an optimal window, so the evidence bore more on whether to resume than on when. Rivaroxaban carried the one clear excess rebleeding signal, favoring apixaban after prior GIB. Individualized risk assessment should guide decisions pending trial data.

## Introduction and background

Atrial fibrillation (AF) is the most common sustained cardiac arrhythmia and a leading contributor to cardioembolic stroke and premature cardiovascular death. Its global prevalence has risen by more than a third over the past two decades and is projected to rise by a further 60% by 2050 as populations age and survive longer after major cardiac events [1]. Patients with AF carry close to a fivefold higher risk of ischemic stroke than those in sinus rhythm, and in those aged 80 and above the arrhythmia becomes the dominant stroke risk factor [2]. These strokes also tend to be more disabling than strokes of other origin [3]. Oral anticoagulation is the mainstay of stroke prevention in this population.

For several decades, vitamin K antagonists were the only oral option. Direct oral anticoagulants (DOACs), comprising the thrombin inhibitor dabigatran and the factor Xa inhibitors apixaban, rivaroxaban, and edoxaban, are now preferred in most guidelines because they offer a more predictable anticoagulant effect without routine monitoring [4]. A pooled analysis of the four pivotal phase 3 trials (RE-LY, ROCKET-AF, ARISTOTLE, and ENGAGE AF-TIMI 48), covering more than 71,000 participants, found that DOACs reduced stroke or systemic embolism and all-cause mortality relative to warfarin and roughly halved intracranial hemorrhage, although gastrointestinal (GI) bleeding (GIB) rose [5].

GIB is the principal safety concern that offsets this benefit. Rates in clinical trials range between 0.35% and 2.09% per year [6]. GI safety, unlike the clear DOAC advantage for intracranial hemorrhage, varies by agent. A meta-analysis of 46 real-world studies ranked rivaroxaban highest for GI hemorrhage and apixaban lowest [7], a pattern a multinational cohort of over 527,000 new users confirmed across all pairwise comparisons [8]. Agent choice therefore matters once a patient has already bled.

Two decisions follow a GI bleed on a DOAC, and they are not the same. The first is whether anticoagulation should be resumed at all, since withholding the drug leaves stroke risk unopposed while restarting may provoke further bleeding before the mucosa has healed. The second, contingent on the first, is the interval after which resumption is safest. Observational cohorts increasingly speak to the first question. The second has proved far harder to characterize, because most studies compared patients who resumed against those who did not, rather than contrasting defined early and late restart windows [9,10].

Guidelines have not closed this gap. The 2020 ACC Expert Consensus Decision Pathway advises considering resumption once hemostasis is secured but gives no DOAC-specific interval [11], and the 2023 American College of Cardiology/American Heart Association/American College of Chest Physicians/Heart Rhythm Society (ACC/AHA/ACCP/HRS) guideline states that data on the timing of DOAC resumption after GIB are sparse and makes no recommendation [12]. In practice, clinicians manage these patients without reliable guidance.

The present review synthesized the observational evidence on DOAC resumption after GIB in adults with AF. It examined whether resumption was associated with recurrent GIB, thromboembolic events, and all-cause mortality, and, separately, whether the available data defined an optimal resumption interval. Because primary studies rarely contrasted early against late windows, the review anticipated that the evidence would weigh more on the first question than the second.

## Review

Methods

Study Design and Registration

This systematic review was conducted and reported in accordance with the Preferred Reporting Items for Systematic Reviews and Meta-Analyses (PRISMA) 2020 guidelines [13]. Eligibility criteria and the extraction form were fixed a priori, but the review was not registered with PROSPERO or any equivalent registry.

PICO Framework and Review Question

The review question was structured using the PICO framework. The population comprised adult patients with atrial fibrillation who experienced GIB while receiving direct oral anticoagulant (DOAC) therapy. The exposure of interest was resumption of anticoagulation after the index GI bleed, together with its timing. The comparator was non-resumption. The outcomes were recurrent GIB, thromboembolic events, and all-cause mortality.

Eligibility Criteria

Studies were selected according to predefined inclusion and exclusion criteria, established before screening commenced. Eligible studies included adults aged 18 years or older with atrial fibrillation as the primary indication for anticoagulation, who experienced GIB as the index event while receiving a DOAC (apixaban, rivaroxaban, dabigatran, or edoxaban). Studies were required to report the timing of anticoagulant resumption after the index bleed and to include at least one of the following clinical outcomes: stroke or systemic embolism, recurrent GIB, major bleeding, or all-cause mortality. Only original human studies published in English were considered. Studies with mixed oral anticoagulant populations were eligible provided DOAC-treated patients constituted a clinically meaningful proportion of the cohort.

Studies were excluded if they enrolled exclusively pediatric populations, if the primary anticoagulation indication was other than atrial fibrillation without a clinically meaningful atrial fibrillation subgroup, or if the index bleeding event was non-GI in origin (including intracranial hemorrhage, hematuria, epistaxis, or postoperative bleeding without a GI source). Studies of warfarin or vitamin K antagonists only were excluded, as were mixed warfarin-DOAC studies in which DOAC-treated patients did not constitute a clinically meaningful proportion of the cohort. Studies that did not report the timing of resumption or did not report clinical outcomes after resumption were excluded.

Where a primary study contributed a non-pooled outcome that the meta-analyses did not capture, it was retained as a standalone included study even if it appeared as a constituent cohort within a meta-analysis for a different outcome. To avoid double-counting in the narrative synthesis, these studies were cited only for the outcomes they uniquely contributed: Sengupta et al. for thromboembolism and Valanejad et al. for mortality. Neither was cited as primary evidence for rebleeding, which was already represented by the pooled estimate from Morarasu et al. [6,14,15].

Records excluded from the primary outcome synthesis were retained as contextual sources where they directly informed interpretation of anticoagulation resumption strategies, evidence gaps, or findings related to GIB in patients with atrial fibrillation receiving DOACs.

Information Sources and Search Strategy

A systematic literature search was conducted across six electronic databases: MEDLINE, PubMed, Scopus, Google Scholar, ScienceDirect, and MDPI. Searches were limited to studies published between January 2015 and April 2026. The January 2015 start was a pragmatic limit. Direct oral anticoagulants had been recommended for non-valvular atrial fibrillation since 2011 [16], yet the first study reporting the review's outcomes in a DOAC-treated population appeared only in 2018 [14]. No gray literature sources, trial registries, or conference proceedings were searched. The search strategy combined terms related to the population (atrial fibrillation), the exposure (direct oral anticoagulants), the clinical event (GIB), and the intervention of interest (resumption or restart of anticoagulation), with Boolean operators applied throughout. For PubMed, Medical Subject Headings (MeSH) terms were incorporated alongside keyword-based Boolean and advanced search strategies; keyword-based Boolean searches were used for all other databases. One additional study was identified through manual screening of reference lists and forward citation tracking. The full electronic search strategies for each database are presented in Table 1.

**Table 1:** Electronic search strategies, databases, and record yields. AF, atrial fibrillation; DOAC, direct oral anticoagulant; GI, gastrointestinal; Majr, MeSH major topic; MDPI, Multidisciplinary Digital Publishing Institute; MeSH, Medical Subject Headings; NOAC, non-vitamin K oral anticoagulation; OAC, oral anticoagulant

Search strategy	Database used	Number of papers identified
("atrial fibrillation") AND ("gastrointestinal bleeding") AND (anticoagulants OR DOAC OR NOAC OR apixaban OR rivaroxaban OR dabigatran OR edoxaban) AND (restart OR resumption OR resume)	EBSCO MEDLINE	24
("gastrointestinal bleeding" OR "gastrointestinal hemorrhage" AND ("atrial fibrillation") AND (DOAC OR NOAC OR apixaban OR rivaroxaban OR dabigatran OR edoxaban OR anticoagulants) AND (restart OR resumption OR resume OR reinitiation)	PubMed (Advanced)	41
("atrial fibrillation") AND ("gastrointestinal bleeding") AND (anticoagulant OR DOAC OR NOAC) AND (restart OR resumption)	PubMed	27
(("Gastrointestinal Hemorrhage"[Majr]) AND "Atrial Fibrillation"[Majr]) AND "Anticoagulants"[Majr]	PubMed (MeSH)	79
( "atrial fibrillation" AND ( "gastrointestinal bleeding" OR "GI bleeding" ) AND ( anticoagulant* OR DOAC OR NOAC ) AND ( restart OR resumption ) )	Scopus	30
"atrial fibrillation" "gastrointestinal hemorrhage" (DOAC OR DOACs OR NOAC OR NOACs OR "direct oral anticoagulant") resumption timing	Google Scholar	300
("atrial fibrillation") AND ("gastrointestinal bleeding") AND (DOAC OR apixaban OR rivaroxaban OR dabigatran OR edoxaban) AND (restart OR resumption)	ScienceDirect	301
atrial fibrillation gastrointestinal bleeding anticoagulant	MDPI	29
	Included manually	1

Study Selection

**Figure 1:**
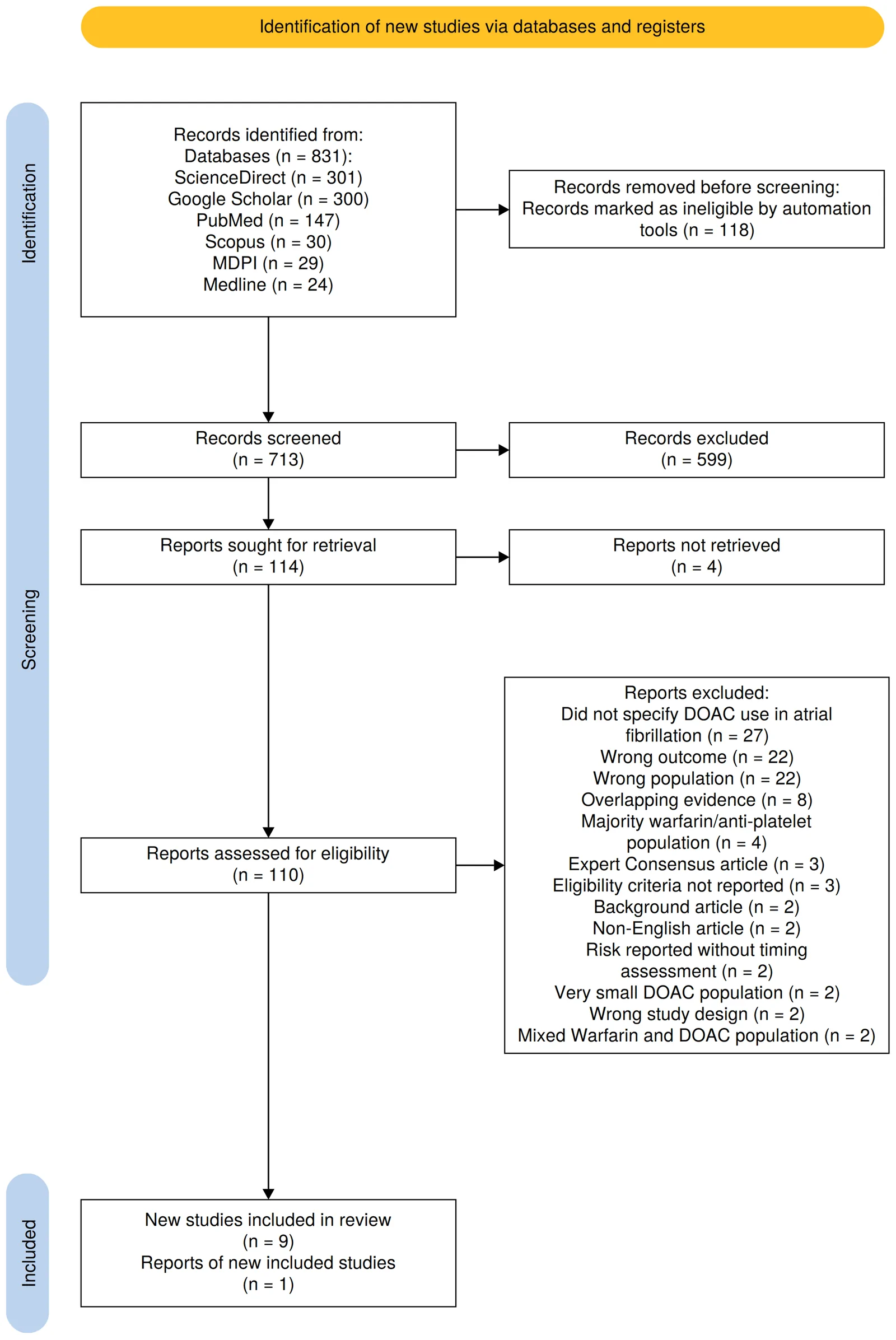
PRISMA 2020 flow diagram depicting the study selection process. DOAC, direct oral anticoagulant; MDPI, Multidisciplinary Digital Publishing Institute; PRISMA, Preferred Reporting Items for Systematic Reviews and Meta-Analyses

All identified records were screened in two sequential stages. Titles and abstracts were reviewed first to exclude records clearly irrelevant to the review question. Records that could not be confidently excluded at that stage proceeded to full-text assessment, where final inclusion decisions were made according to the predefined eligibility criteria. The study selection process was documented using a PRISMA 2020 flow diagram (Figure 1). Duplicate records were removed before screening commenced.

Titles, abstracts, and full texts were screened independently by two reviewers. Disagreements were resolved through discussion; where consensus could not be reached, a third independent reviewer adjudicated. Screening was supported by Rayyan AI [17], which was used for duplicate detection and collaborative title-abstract screening. When multiple publications appeared to originate from overlapping cohorts, each article was evaluated individually according to its study design, patient population, exposure definition, and reported outcomes to avoid duplicate inclusion of the same patient dataset.

Data Extraction

Data were extracted using a pilot-tested extraction form developed before the search was conducted. One reviewer completed the extraction and a second reviewer independently verified all extracted data. Disagreements were resolved by discussion, with recourse to a third reviewer when necessary. The following variables were extracted from each included study: author and publication year; country; study design; sample size; anticoagulation indication; timing of DOAC resumption; rebleeding, thromboembolic, and mortality outcomes; duration of follow-up; reported study limitations; and study conclusion. Quantitative data, including hazard ratios (HRs), odds ratios (ORs), confidence intervals (CIs), *P*-values, and event rates, were extracted wherever available. Extracted data were organized according to timing of DOAC resumption and clinical outcome to support the narrative synthesis.

Risk-of-Bias Assessment

Risk of bias was assessed independently by two reviewers using validated tools appropriate to each study design. Systematic reviews and meta-analyses were assessed using the Assessment of Multiple Systematic Reviews (AMSTAR 2) tool, version 2 [18]. Observational cohort studies were assessed using Risk of Bias in Non-randomized Studies of Interventions, version 2 (ROBINS-I V2) [19]. The single included case report was appraised using the Joanna Briggs Institute Critical Appraisal Checklist for Case Reports (JBI) [20]. Disagreements between reviewers were resolved through consensus discussion or, where necessary, consultation with a third reviewer.

Data Synthesis

A meta-analysis was not performed, given anticipated heterogeneity in study designs, patient populations, outcome definitions, and the variable thresholds used to define early versus delayed anticoagulant resumption across the included studies. Findings were synthesized narratively. Studies were grouped by design and outcomes, and results were summarized descriptively, with attention to the timing of DOAC resumption and the association of resumption with thromboembolic events, recurrent bleeding, and all-cause mortality. Effect measures reported in individual studies, including HRs, ORs, and their 95% CIs, were presented as extracted without statistical pooling. Publication bias and certainty of evidence were not assessed, as the narrative synthesis approach and the heterogeneity of included study types made formal assessment inappropriate. This is acknowledged as a limitation of the present review.

Ethics and Data Availability

This systematic review used published data exclusively and did not involve direct recruitment of human participants. Ethical approval was therefore not required. All data used in the review were derived from peer-reviewed articles included in the final evidence synthesis. No primary data were collected, held, or analyzed beyond what was reported in the included publications. Data extraction forms are available from the corresponding author on reasonable request. 

Results

Study Selection

The search across six databases returned 831 records: ScienceDirect (n = 301), PubMed (n = 147), Google Scholar (*n* = 300), Scopus (*n* = 30), MDPI (*n* = 29), and MEDLINE (*n* = 24). Manual reference-list review identified one additional study, yielding 832 records. Rayyan AI was used for deduplication and prescreening; automation removed 118 records, leaving 713 for title and abstract screening, of which 599 were excluded. Full-text retrieval was sought for 114 reports; four could not be obtained, and 110 were assessed for eligibility. Of these, 101 were excluded. Nine studies were included from database searching and one from manual review, yielding a total of 10 studies (PRISMA 2020 flow diagram; Figure 1).

Characteristics of Included Studies

The 10 studies were published between 2018 and 2025 and enrolled 68,611 patients (Table 2). Atrial fibrillation accounted for 71.9% to 100% of patients; the remainder were anticoagulated for venous thromboembolism (6.7% to 20.1%) or prosthetic heart valves (12.3%). The anticoagulants included apixaban, rivaroxaban, dabigatran, and edoxaban. Mean or median age ranged from 74 to 82 years, and follow-up ranged from 1 month to 3.1 years.

**Table 2:** Characteristics of included studies. Summary of study design, patient population, anticoagulation indication, and methodological limitations across the 10 included studies. AC, anticoagulation; AF, atrial fibrillation; DOAC, direct oral anticoagulant; GIB, gastrointestinal bleeding; MA, meta-analysis; NVAF, nonvalvular atrial fibrillation; obs., observational; SR, systematic review; VKA, vitamin K antagonist; VTE, venous thromboembolism; OTC, over-the-counter; NSAID, nonsteroidal anti-inflammatory drug; ICD-9, International Classification of Diseases, Ninth Revision

Author (Year)	Design and country	Purpose	Number/Age/Follow-up	AC Indication	Limitations
Morarasu et al. (2024) [6]	SR + MA of 5 obs. studies; United States and Japan	Compare outcomes, especially rebleeding, in patients who restarted versus stopped DOACs after GIB.	N = 2,837 on DOACs (1,290 resumed/1,547 stopped); mean age 77.2/77.9 years; follow-up 3-13.2 months	Mostly AF	Only 5 small, retrospective studies included. Inconsistent patient criteria and study endpoints across studies. No raw data available for subgroup analysis. Claims databases lacked follow-up and adherence data. Publication bias present; trim-and-fill analysis unreliable with fewer than 10 studies.
Hu et al. (2022) [21]	Network MA of 9 obs + 1 post hoc; South Korea, Sweden, Italy, United States, Denmark, Latin America	Assess the safety and effectiveness of resuming DOACs versus VKAs in AF patients with a prior GIB.	N = 59,244 AF; 27,793 resumed DOACs; 6,816 did not resume AC; follow-up 1 month to 3.1 years	NVAF	All included studies were observational. Registry/insurance databases could not confirm medication adherence. Optimal timing for restarting anticoagulation still unclear; prospective trials needed.
Morarasu et al. (2025) [22]	Single-center retrospective cohort; Romania	Evaluate rebleeding events and their link to stopping versus continuing DOACs after an index GI bleed.	N = 120 on DOACs; resumed = 91 (75.8%), held = 29; median age 75 years; follow-up 15.2 months	AF 90.8%; VTE 6.7% AF; VTE 2.5%	Small single-center cohort; many patients excluded due to data gaps. Anticoagulation decisions left to physician discretion, not protocol. Bleeding severity and clinician preference may have influenced decisions. Medication adherence and pre-event PPI use could not be reliably assessed.
Gennaro et al. (2024) [23]	Retrospective population-based cohort; Italy	Measure how restarting versus withholding anticoagulation affects stroke recurrence, major bleeding, and mortality in AF patients on DOACs who experienced an anticoagulation-related event.	N = 1,029 on DOACs; 607 major bleeds (half GIB); mean age 77 years; follow-up median 1.38 years	Non-valvular AF (DOAC-naïve)	Retrospective, administrative data adherence based on prescriptions, not actual intake; 120-day blanking period was arbitrary. Residual confounding possible despite propensity score adjustment. No specific timing guidance; major bleeding included both GI and non-GI sources.
Candeloro et al. (2021) [9]	Multicenter retrospective cohort; Italy, the Netherlands, Canada	Examine rates of rebleeding, thromboembolism, and death in patients who experienced GI bleeding on oral anticoagulants, and how timing of resumption affected these outcomes.	N = 948; 417 on DOACs; mean age 78.9 years; follow-up two years	AF 81.6%; VTE 15.9%; Valve 12.3%	Retrospective; resumption decisions made by physicians, not protocol. Confounding by indication likely (sicker patients more often discontinued). Antiplatelet use and adherence not fully tracked. Too few events for reliable subgroup analyses; residual confounding cannot be excluded. Risk of immortal time bias (partially addressed by time-dependent analysis)
Little et al. (2021) [24]	Population-based cohort; Ontario, Canada	Describe OAC prescribing patterns after OAC-related bleeding and compare rates of rebleeding, thrombosis, and death between patients who resumed versus those who did not.	N = 6,793 (4,297 GIB); 2,919 DOACs; median age 82 years; follow-up median 82 weeks	AF 81.8%; VTE 20.1%	Residual confounding possible; causes of death could not be determined. Only hospital-level bleeding events captured; severity not further defined. OTC aspirin and NSAID use not fully accounted for; adherence assumed from prescriptions. 100-day blanking period excluded early events. Limited to adults aged 66 and over; outcomes by resumption timing could not be assessed.
Yanagisawa et al. (2021) [25]	Single-center retrospective cohort; Japan	Clarify rebleeding and thrombotic event rates in DOAC-treated NVAF patients after bleeding and examine the role of resuming versus stopping anticoagulation.	N = 96 NVAF with bleeding (59% GIB); median age 76 years; follow-up median 2.3 years	NVAF	Small single-center retrospective study. No comparison of different resumption timing windows. Causality cannot be established; very wide confidence intervals limit reliability.
Valanejad et al. (2020) [15]	Single health-system retrospective cohort; United States	Examine prescribing patterns and clinical outcomes in patients who experienced a GIB while taking a DOAC. The primary endpoint was readmission for recurrent GIB within 90 days of discharge.	N = 57 on DOACs (37 (64.9%) stopped/18 (31.6%) resumed within 7 days)); median age 75/74 years; 90 days recurrent GIB, 12 months mortality	AF 71.9%; PE 15.8%; DVT 14%; Off label 3.5%	No comparable published cohort existed at the time of analysis. Small sample size led to inadequate power to detect clinically meaningful differences. Results came from a single institution, limiting external applicability. Chart-based data collection introduced a risk of documentation error & misclassification of patients into groups.
Sengupta et al. (2018) [14]	Retrospective cohort; United States	Examined DOAC refill patterns following GIB hospitalization and the associated risks of recurrent bleeding and thromboembolism. Primary endpoints were 90-day readmissions for recurrent GIB and thromboembolic events.	N = 1,338; 586 (44%) resumed, 752 withheld; Median age 78/79 years; 90 days and 6 months post-discharge	NVAF	No mortality analysis. ICD-9 codes used to identify events had not been validated in this database, and bleeding source rested on billing data rather than endoscopy. Minority may have been anticoagulated for other reasons. Patient switched to warfarin counted as non-resumers, which might underestimate true restart rate. Low absolute event counts raised the risk of model overfitting; covariates were restricted to one per ten outcome events to partially address this. Pharmacy claims filed with a delay after the actual restart date could further underestimate early resumption. Claims cannot capture events that did not result in readmission, so event rates may be underestimated.
Riario Sforza et al. (2018) [26]	Case report; Italy	Report the use of idarucizumab to reverse dabigatran-related major GI bleeding and describe the safety and timing of restarting dabigatran afterward.	N = 1 (87-year-old woman, dabigatran 110 mg BID, CHA₂DS₂-VASc 5); follow-up 282 days	Chronic AF	Single-patient case report; no comparative data. Resumption timing driven by patient/family consent, not standardized criteria. Potential funding bias (Boehringer Ingelheim Italy)

Risk-of-Bias Assessment

**Figure 2:**
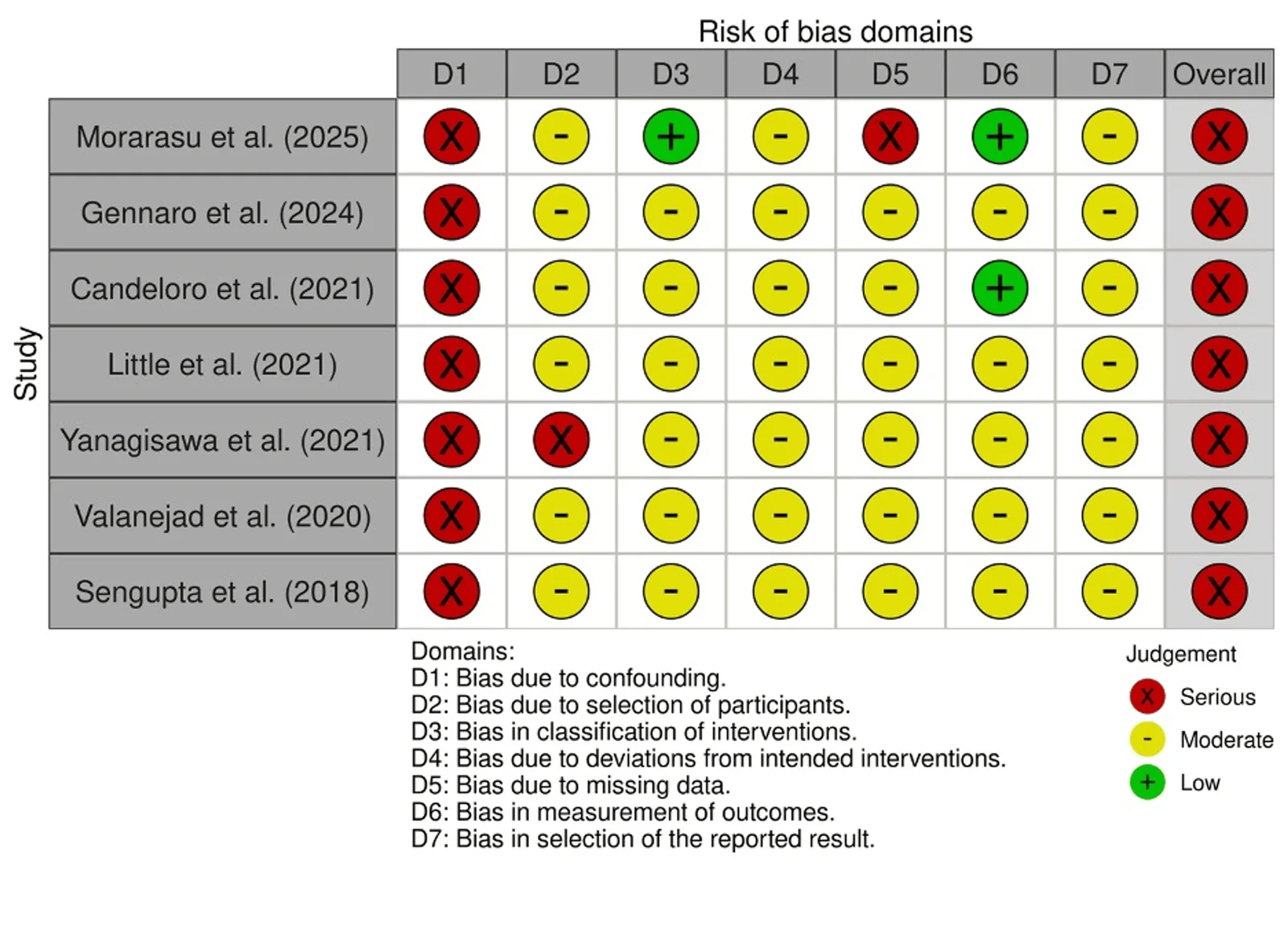
Traffic-light summary of methodological quality for non-randomized studies included in the review. Adapted from the Risk Of Bias In Non-randomized Studies of Interventions, Version 2 (ROBINS-I V2) [19]. Green indicates low risk, yellow indicates moderate risk, and red indicates serious risk. The figure was generated using robvis [27] and accessed on May 30, 2026. The included studies are Candeloro et al. [9], Sengupta et al. [14], Valanejad et al. [15], Morarasu et al. [22], Gennaro et al. [23], Little et al. [24], and Yanagisawa et al. [25].

AMSTAR-2 rated Morarasu et al. [6] as critically low and Hu et al. [21] as low (Table 3). ROBINS-I V2 assessed the seven observational studies across seven domains (Figure 2): confounding (D1) was a concern in all seven studies, and participant selection (D2) was a concern in the claims-based studies. Overall risk of bias ranged from moderate to serious, with none of the studies rated as low risk across all domains. The JBI checklist rated the case report by Sforza et al. [26] as 8 of 8 (Table 4).

**Table 3:** Assessment of Multiple Systematic Reviews (AMSTAR 2), version 2. NRSI, non-randomized studies of interventions

AMSTAR 2 item	Morarasu et al. [6]	Hu et al. [21]
1	Yes	Yes
2	Partial Yes	Partial Yes
3	No	Yes
4	No	No
5	Yes	Yes
6	Yes	Yes
7	No	No
8	Partial Yes	Yes
9	Yes (NRSI)	Partial Yes (NRSI)
10	No	No
11	Yes (NRSI)	Yes (NRSI)
12	No	No
13	Yes	Yes
14	Yes	Yes
15	Yes	Yes
16	No	Yes
Overall rating	Critical low	Low

**Table 4:** JBI critical appraisal checklist for case reports. JBI, Joanna Briggs Institute Source: [26].

Items	Response
1. Were the patient’s demographic characteristics clearly described?	Yes
2. Was the patient’s history clearly described and presented as a timeline?	Yes
3. Was the current clinical condition of the patient on presentation clearly described?	Yes
4. Were diagnostic tests or assessment methods and the results clearly described?	Yes
5. Was the intervention(s) or treatment procedure(s) clearly described?	Yes
6. Was the post-intervention clinical condition clearly described?	Yes
7. Were adverse events (harms) or unanticipated events identified and described?	Yes
8. Does the case report provide takeaway lessons?	Yes
Overall	8

Reported Definitions and Distribution of Resumption Timing

All 10 studies reported when anticoagulation was restarted; the intervals were wide, from within seven days to several months (Table 5). Early restarts clustered in the single-center cohorts. Yanagisawa et al. saw about 80% resume within seven days and roughly 90% within 14 days [25]. Valanejad et al. defined resumption as restart within seven days, and recorded 31.6% resuming [15]. Morarasu et al. reported a similar pattern, with 73.6% of the patients back on treatment within seven days [22]. Candeloro et al. sat at the early end too, with a median of six days among 66.4% who restarted [9]. Later restarts were the norm in the larger administrative cohorts. Little et al. reported a median of 24 days, with 53% back on treatment within 30 days and 71.6% within a year [24], while Sengupta et al. reported a median refill at 40 days, with 18% refilling within 30 days and 44% over six months [14]. The two meta-analyses spanned both ends: the cohorts pooled by Morarasu et al. ranged from seven days to 45 days, with Tapaskar 2021 unrecorded [6], and the datasets in Hu et al. used resumption thresholds running from within 30 days to 3.1 years [21]. No included study set a predefined early window against a predefined late one. Candeloro et al. came closest, using a time-dependent analysis, and found that recurrent bleeding rose after restart, whatever the timing of resumption [9]. Two administrative cohorts applied blanking periods, 100 days in Little et al. [24] and 30 and 120 days in Gennaro et al. [23], but these excluded early events to limit immortal-time bias and were never intended to compare early with late resumption. The remaining studies reported when patients resumed without testing whether timing changed outcomes.

**Table 5:** Clinical outcomes by study. Reported estimates for rebleeding, thromboembolism, and all-cause mortality associated with DOAC resumption versus non-resumption after GIB, along with each study’s overall conclusion and timing of resumption. *Statistically significant (*P* < 0.05). AF, atrial fibrillation; CI, confidence interval; DOAC, direct oral anticoagulant; GIB, gastrointestinal bleeding; HR, hazard ratio; MACCE, major adverse cardiac and cerebrovascular events; NS, not statistically significant; OR, odds ratio; IQR, interquartile range; VTE, venous thromboembolism

Author (Year)	Timing of Resumption	Rebleeding	Thromboembolism	Mortality	Conclusion
Morarasu et al. (2025) [6]	Range: ≤7 days to 45 days across cohorts	DOAC vs. no resumption: OR 1.09 (95% CI 0.77-1.53), *P* = 0.632; (NS)	No pooled estimate; individual cohorts suggest possible benefit	No pooled estimate; authors suggest possible benefit	Rebleeding similar; overall benefit of resumption suggested
Hu et al. (2022) [21]	≤30 days to 3.1 years across cohorts	DOAC vs. no resumption: HR 1.22 (0.88-1.71) (NS); Rivaroxaban: HR 1.67 (1.16-2.65)*	DOAC vs. no resumption: HR 0.69 (0.40-1.20) (NS)	DOAC: HR 0.57 (0.40-0.84)*	DOACs safer than warfarin; rivaroxaban should be avoided
Morarasu et al. (2025) [22]	≤7 days 73.6%; 7-14 days; >14 days	HR 1.229 (0.494-3.054) (NS); Prior GIB HR 4.070 (1.771-9.354)*	Not reported directly	Not reported directly	Timing and agent not significant; prior GIB main risk factor
Gennaro et al. (2024) [23]	Within 120-day blanking period; plateau at 120 days	Part of composite; major bleeding HR 0.51 (0.24-0.68)* at 120-day blanking period, HR 0.42 (0.31-0.57)* at 30-day blanking period	Part of composite	Part of composite	Resumption reduces composite events regardless of index event type (ischemic stroke, intracranial hemorrhage, or major bleeding)
Candeloro et al. (2021) [9]	Median 6 days (range 1-447); 66.4% resumed	Adjusted HR 1.55 (1.08-2.22)*; Independent of timing	Adjusted HR 0.34 (0.21-0.55)*	Adjusted HR 0.50 (0.36-0.68)*	Lower thromboembolism and death; higher rebleeding; patient factors matter more than timing
Little et al. (2021) [24]	Median 24 days (IQR 9-60); 71.6% resumed within 365 days	Adjusted HR 2.02 (1.69-2.40)*; 64.5% recurrent GI	Adjusted HR 0.56 (0.44-0.71)*	Adjusted HR 0.54 (0.47-0.62)*	Resumption increases rebleeding but reduces thrombosis and mortality; individualized approach recommended
Yanagisawa et al. (2021) [25]	~90% resumed within 14 days; 80% within 7 days	All 11 rebleeds in resumed patients; HR 9.6×10⁸ (3.7×10⁸-24.1×10⁸)*	All 4 systemic thromboses in withheld patients; HR 3.24 (0.37-28.62) (NS, small number)	2 cardiac deaths; MACCE occurred in 6 patients total (4 systemic thromboses, 2 cardiac deaths), with 4 of 6 (66.7%) having withheld anticoagulation	Rebleeding high in resumed; thrombosis high in withheld; prior GIB key predictor
Valanejad et al. (2020) [15]	31.6 % resumed within 7 days; 64.9% withheld; 3.5% switched to warfarin	90-day recurrent GIB readmission: withheld 2.5%, resumed 5.6%, P= 0.83 (NS)	Not reported	12-month all-cause mortality: withheld 10.8%, resumed 22.2%, switched to warfarin 0, *P* = 0.28 (NS)	Early DOAC restart is safe. Rivaroxaban predominated pre-admission for GIB. Major bleeds favored withholding. Minor source bleeds supported early resumption.
Sengupta et al. (2018) [14]	247 (18%) refilled within 30 days, 586 (44%) refilled within 6 months, 752 withheld	At 90 days: 30-day refill HR 1.44 (95% CI 0.72-2.68), *P* = 0.29 (NS). At 60 days: 30-day refill: HR 1.56 (95% CI 0.95-2.47), *P* = 0.08 (NS)	At 90 days: 30-day refill: HR 0.98 (95% CI 0.37-2.21), *P* = 0.96 (NS)	Not reported	DOAC restart did not raise rebleeding or thromboembolism risk. Older, sicker patients were less likely to be restarted. Rivaroxaban carried the highest rebleeding risk; prior VTE and thienopyridine use drove thromboembolism and rebleeding, respectively. Early resumption considered safe overall.
Sforza et al. (2018) [26]	~20 days post-bleed (delayed by consent)	None during follow-up	None during follow-up	Alive at 282 days	Dabigatran safely restarted post-idarucizumab reversal; larger studies needed.

Recurrent GIB

Rebleeding was the most widely reported outcome, and the pooled estimate found no overall difference between resumption and discontinuation. Morarasu et al. reported an OR of 1.09 (95% CI 0.77-1.53; *P* = 0.632; I2 = 0%), which trim and fill adjusted to 1.06 (95% CI 0.76-1.49) after Egger's test flagged publication asymmetry (*P* = 0.02); absolute rebleeding among resumers ranged from 3.5% [14] to 18.8% [6], and thienopyridine use raised the 90-day risk (HR 3.12, 95% CI 1.55-5.81). Two cohorts recorded a higher rebleeding rate with resumption. Little et al. reported an adjusted HR of 2.02 (95% CI, 1.69-2.40), with the GI tract being the source of 64.5% of rebleeds [24]. Candeloro et al. reported an adjusted HR of 1.55 (95% CI, 1.08-2.22), with landmark estimates of 1.52, 1.63, and 1.99 at days 7, 14, and 21, respectively. Previous bleeding, index major bleeding, and lower glomerular filtration rate were each associated with a higher risk of rebleeding [9]. The agent mattered more than resumption itself: Hu et al. found no association at the class level (HR 1.22, 95% CI 0.88-1.71) but a higher risk with rivaroxaban (HR 1.67, 95% CI 1.16-2.65) that was absent for apixaban, dabigatran, and edoxaban [21]. Patient history also mattered; in a study by Morarasu et al., a prior GI bleed was associated with an HR of 4.07 over 15 months (95% CI 1.77-9.35; *P* = 0.001), compared with an overall resumption HR of 1.23 (95% CI 0.49-3.05) [22]. The smaller cohorts observed few events. Yanagisawa et al. recorded rebleeding in 11 of 96 patients (4.5% per year), 10 of whom had GIB, with prior GIB again predictive (*P* = 0.024) [25]. Gennaro et al., among 607 patients with an index major bleed, reported an adjusted HR of 0.51 (95% CI 0.24-0.68; 120-day blanking) for a composite of death, stroke, intracranial hemorrhage, and major bleeding, with no change associated with a switch in agent or dose (*P* = 0.52) [23]. Sforza et al. recorded no rebleeding over 282 days [26].

Thromboembolism

Across the cohorts that measured it, three reported a statistically significant reduction in thromboembolic events with resumption. Candeloro et al. recorded 77 events (8.1%) over a mean of 175 days, with cumulative incidence rising from 2.36% (95% CI 1.52-3.49) at 30 days to 7.42% (95% CI 5.75-9.36) by study end; on-treatment status was associated with an adjusted HR of 0.34 (95% CI 0.21-0.55), with landmark estimates of 0.16, 0.16, and 0.15 at days 7, 14, and 21, respectively [9]. Little et al. reported an adjusted HR of 0.56 (95% CI 0.44-0.71), consistent across DOAC and warfarin subgroups [24]. In three other cohorts, the reduction did not reach statistical significance. In Yanagisawa et al., MACCE occurred in six of 96 patients (2.5% per year), and anticoagulation had been withheld in four of them, including all four systemic thrombotic events (adjusted HR 3.24, 95% CI 0.37-28.62; *P* = 0.290) [25]. Sengupta et al. found no significant 90-day reduction with a refill within 30 days (HR 0.98, 95% CI 0.37-2.21), although prior venous thromboembolism predicted events (HR 3.30, 95% CI 1.29-7.38) [14]. Hu et al. found no reduction at the class level (HR 0.69, 95% CI 0.40-1.20), although its Asian subgroup showed a reduction versus warfarin (HR 0.61, 95% CI 0.54-0.68) [21].

All-Cause Mortality

Mortality was reported across cohorts of every size, and the larger ones consistently recorded lower death rates with resumption. Candeloro et al. documented 185 deaths (19.5%) over a median of 118 days, with on-treatment status associated with an adjusted HR of 0.50 (95% CI 0.36-0.68) and landmark estimates of 0.30, 0.35, and 0.35 at days 7, 14, and 21 [9]. Little et al. reported an adjusted HR of 0.54 (95% CI 0.47-0.62) [24]. Hu et al. found a reduction in mortality with DOAC resumption compared with no anticoagulation (HR 0.57, 95% CI 0.40-0.84) [21]. The smaller cohort showed the opposite trend: Valanejad et al. recorded 12-month mortality in four of 37 held patients (10.8%), four of 18 resumers (22.2%), and none of the two who switched to warfarin (P = 0.28) [15]. Yanagisawa et al. recorded two cardiac deaths among their six MACCE patients, four of whom had withheld anticoagulation [25]. Morarasu et al. did not report mortality [6,22], and Sforza et al. reported survival at 282 days [26].

Discussion

The evidence answered one question far more clearly than the other. On whether to resume a DOAC after GIB, the included cohorts pointed consistently in the same direction. On when to resume, they were largely silent because almost none compared a defined early window against a defined late one.

Whether to Resume

Two findings defined this question. Resumption was not associated with more recurrent bleeding at the drug-class level, since neither included meta-analysis showed an increase [6,21]. Two cohorts did record more rebleeding among resumers [9,24], and in the smallest cohort every rebleed occurred in a resumer, although only eleven patients had resumed [25]. Against that, resumption was associated with fewer thromboembolic events in the cohorts that measured them directly [9,24] and with lower mortality in the largest datasets, including the Hu et al. meta-analysis [21]. The direction is biologically coherent, since atrial fibrillation sustains a prothrombotic state and any interval off anticoagulation leaves stroke risk unopposed [28]. Gennaro et al. found the same for a composite endpoint, and the benefit held when the agent or dose was switched, which located it in the act of resuming rather than in the regimen chosen [23].

These associations should be read with caution. Sicker patients were the ones clinicians tended not to restart, and patients had to survive long enough to be restarted, so confounding by indication and immortal time both push resumption to look more protective than it is. One small single-center cohort ran the other way on mortality, but the difference was not significant and rested on a handful of events [15]. Two of the cohorts showing benefit also included warfarin patients, so their results could not be attributed to DOACs alone [9,24]. The protective signal came from the larger, better-adjusted studies and the discordant signals from the smallest, which is why the balance of evidence favored resumption without settling the question.

When to Resume

Direct evidence on timing was thin, and the reason lay partly in what drove the outcomes. Rebleeding depended on the lesion and the patient rather than on the day of restart. A prior gastrointestinal bleed predicted recurrence across three cohorts and was the single strongest risk factor in Morarasu et al. [9,22,25], impaired renal function compounded it [29], and concurrent thienopyridine therapy tripled the 90-day risk, so withdrawing the antiplatelet did more than delaying the anticoagulant [14]. Sengupta et al. pointed in the same way for thromboembolism, since a refill within 30 days did not lower it, whereas prior venous thromboembolism more than tripled it, again placing the risk in the patient rather than in the timing [14]. Where timing was examined within a study, it carried little weight, and no cohort contrasted a prespecified early window against a late one; the thresholds used elsewhere were too different to combine. A microsimulation by Pappas et al. placed the best restart at about a month for apixaban and six weeks for warfarin, earlier when stroke risk was high [30], but those were modeled figures, and since no fixed window emerged from the observational data, their precision should not be overread. Current atrial fibrillation guidance offers no restart interval [12,31], and the present data support anchoring the decision to the patient's stroke and bleeding risk and to the agent rather than to a set delay.

Agent Selection

One agent stood apart. Rivaroxaban was the only DOAC with a clearly higher rebleeding risk, and that risk disappeared once the agents were pooled as a class. A meta-analysis by Barakakis et al. ranked rivaroxaban worst for gastrointestinal hemorrhage [7], and the Lau et al. cohort found apixaban the safest in every comparison [8]. A plausible mechanism sits behind this: a share of any DOAC dose reaches the intestinal lumen unabsorbed and acts locally on the mucosa, an effect amplified by P-glycoprotein efflux in the distal small bowel, so luminal exposure is a class property [32]. Rivaroxaban most likely fares worst because its once-daily dosing produces higher peak concentrations than the twice-daily agents, although this does not fully explain the gap [33].

Clinical Implications

Permanent discontinuation after a bleed is hard to justify when stroke risk is high. Once hemostasis is secure and the source treated, the remaining choices are agent and dose: European guidance lists dabigatran 110 mg twice daily as a lower-intensity option [34], and rechecking renal function with dose reduction and adding a proton pump inhibitor after a prior ulcer are reasonable [29]. Reversal followed by a deliberate restart is feasible: a single case here reversed dabigatran with idarucizumab and restarted it without further bleeding [26], and andexanet alfa offers an analogous route for the factor Xa inhibitors, although no included study tested it [35]. A recent cohort added support, with a DOAC outperforming warfarin for subsequent major bleeding and net clinical outcome among patients who resumed within 90 days [36]. What remains absent is a trial on timing; NOAC-GAP (NCT03785080) may provide the first randomized answer, and until then, the observational data are the best guide.

Limitations

Several limitations apply. Every study was observational, and the principal problem is confounding by indication, since clinicians tend to withhold anticoagulation from sicker, frailer patients already at higher risk of death, which makes resumption appear more beneficial than it is. Some cohorts overlapped, so the patient totals count records, not separate people. Definitions of early and delayed resumption differed, follow-up varied, and most studies inferred drug exposure from dispensing records rather than confirmed intake, which biases results toward the null. Some of the studies that disagreed were small enough that a few extra events would have flipped them. Thromboembolism and mortality could not be pooled, the certainty of evidence was not graded, and almost all the data came from North America, Japan, and Western Europe, so generalizability to other settings is limited. No gray literature was searched, which left the review more exposed to publication bias. The search did not include Embase or the Cochrane Central Register of Controlled Trials, and three sources searched (Google Scholar, ScienceDirect, and MDPI) are search engines or publisher platforms rather than curated bibliographic databases, which may have limited retrieval.

## Conclusions

Resuming a DOAC after GIB in AF was associated with fewer thromboembolic events and fewer deaths, with no class-level rise in recurrent bleeding. The patients who bled again were identifiable from their history, and rivaroxaban carried the one clear excess rebleeding risk, so the choice of agent was itself consequential. Direct comparisons of early versus delayed resumption were too few to define an optimal interval. Until a trial settles that question, the evidence favored deliberate resumption, judged by each patient's stroke and bleeding risk, over default permanent discontinuation.
